# ECA-ModNet: a parameter-efficient network for unsound wheat kernel classification

**DOI:** 10.3389/fpls.2026.1902739

**Published:** 2026-08-19

**Authors:** Ming Chen, Yuan Ning, Xiaobo Wen, Teng Li, Pengtao Lv, Zelong Liu, Chengxin Cai

**Affiliations:** 1 School of Artificial Intelligence, Tongling University, Tongling, China; 2 Key Laboratory of Grain Information Processing and Control (Henan University of Technology), Ministry of Education, Zhengzhou, China; 3 College of Software Engineering, Zhengzhou University of Light Industry, Zhengzhou, China; 4 School of Food and Health, Beijing Technology and Business University, Beijing, China

**Keywords:** agricultural computer vision, channel attention, feature modulation, parameter-efficient network, unsound wheat kernel classification

## Abstract

**Introduction:**

Accurate classification of unsound wheat kernels is important for automated grain quality assessment, but improved recognition performance often comes at the cost of increased model complexity.

**Methods:**

This study presents ECA-ModNet, a parameter-efficient convolutional network derived from EfficientNetV2-S. The architecture replaces two early-stage Fused-MBConv blocks with Mod-FusedMBConv blocks to introduce input-dependent local contextual modulation and replaces the squeeze-and-excitation modules in later stages with efficient channel attention to model local cross-channel interactions using fewer attention-related parameters. Experiments were conducted on the seven-class G600 wheat subset of the GrainSpace dataset.

**Results:**

Across three independent runs, ECA-ModNet achieved an accuracy of 90.17 ± 0.21% and a macro-F1 score of 90.23 ± 0.21%, improving upon EfficientNetV2-S by 3.80 and 3.84 percentage points, respectively. The parameter count decreased from 20.19M to 16.49M, while FLOPs increased marginally from 2.90G to 2.95G. ECA-ModNet achieved accuracy statistically comparable to that of ConvNeXt-Tiny and InceptionNeXt-T while using substantially fewer parameters, and obtained 3.03–4.55 percentage points higher mean accuracy than six lightweight baselines.

**Discussion:**

Ablation experiments identified two Stage 1 Mod-FusedMBConv blocks with a 3×3 context kernel as the configuration with the highest mean accuracy among those evaluated. These results indicate that ECA-ModNet offers a favorable accuracy–parameter trade-off for image-based classification of unsound wheat kernels.

## 1 Introduction

Wheat is one of the most important staple crops worldwide. Compared to other major cereal crops, wheat requires relatively less water while being rich in carbohydrates, proteins, and various trace elements, making it a key ingredient in numerous high-value processed foods. Due to its broad adaptability and high nutritional value, wheat serves as a vital food source worldwide and also plays a crucial role in biofuel and pharmaceutical industries (Igrejas and Branlard, 2020).

However, during the growth, harvesting, and storage of wheat, unsound grains frequently occur, including deformed, pest-damaged, and moldy grains. These unsound grains not only degrade the overall quality of wheat but may also diminish its nutritional value and compromise food processing safety. If unsound grains are not effectively identified and separated, they can lead to a decline in wheat yield, negatively impacting agricultural economies and energy supply (Curtis and Halford, 2014). Moreover, wheat grown from unsound grains may suffer from the loss of essential nutrients, posing risks to human health. For instance, the presence of natural antioxidants in wheat influences its potential health benefits in reducing the risk of cancer and coronary heart disease (Yu et al., 2004). Therefore, accurately identifying unsound wheat grains and optimizing seed cultivation systems are essential for ensuring global food and energy security as well as improving human health (Chaves et al., 2013; Fischer, 2008).

Conventional inspection of unsound wheat kernels relies primarily on manual visual assessment. In routine quality inspection, trained personnel classify kernels according to visible characteristics such as color, texture, shape, and surface damage. However, due to subjective bias, even experienced experts often achieve low classification accuracy when relying solely on visual inspection, and the process is both time-consuming and labor-intensive. To overcome the inefficiencies and accuracy limitations of manual methods, many researchers have proposed automatic identification techniques based on traditional feature engineering. These methods rely on manually designed feature extraction strategies to obtain key attributes—such as color, texture, and morphology—from images or spectral data, which are then used for classification or regression analysis. These methods provide more standardized measurements than unaided visual inspection but remain dependent on the quality and transferability of handcrafted descriptors. Their performance depends strongly on handcrafted descriptors, and they cannot learn task-specific representations directly from data. As a result, they struggle to generalize well when dealing with variations in wheat grains under different environmental conditions. Additionally, the manual feature extraction process is inherently subjective, further impacting the stability and consistency of the models.

The emergence of deep CNNs, exemplified by AlexNet (Krizhevsky et al., 2017), shifted image classification from handcrafted descriptors toward end-to-end representation learning. Compared to traditional feature engineering methods, CNNs learn hierarchical nonlinear representations directly from images and can capture subtle differences in kernel color, texture, and morphology. Nevertheless, conventional convolutional blocks primarily aggregate information within local neighborhoods, which may limit flexible input-dependent interaction across spatial regions.

Vision transformers have achieved strong performance in image classification, object detection, and semantic segmentation (Dosovitskiy et al., 2020). By modeling pairwise token interactions, vision transformers provide a flexible mechanism for capturing long-range dependencies. Their success is largely attributed to the Self-Attention mechanism. Unlike an individual convolutional operation, which aggregates information within a local neighborhood, standard self-attention explicitly models pairwise interactions among tokens across spatial positions. This capability has motivated their application to fine-grained agricultural image recognition. However, the quadratic complexity and large memory footprint of self-attention can limit its use in resource-constrained inspection systems.

In practical wheat quality inspection, model efficiency should be evaluated from multiple perspectives, including parameter count, arithmetic complexity, inference latency, and memory usage. Parameter reduction alone does not necessarily translate into faster or more memory-efficient execution. This study therefore focuses on improving the accuracy–parameter trade-off of EfficientNetV2-S and evaluates its computational and runtime characteristics separately.

To this end, we propose ECA-ModNet, a parameter-efficient network based on EfficientNetV2-S (Tan and Le, 2021). Mod-FusedMBConv is introduced into two compatible blocks in the early stage to provide input-dependent local contextual modulation. In the later stages, ECA (Wang et al., 2020) replaces SE to reduce attention-related parameters while preserving cross-channel interaction. The resulting architecture is evaluated in terms of classification performance, parameter count, FLOPs, desktop CPU/GPU runtime, throughput, peak GPU memory, and energy consumption. Embedded inference was additionally benchmarked on an NVIDIA Jetson Orin Nano Super using FP16 TensorRT.

The main contributions are summarized as follows:

1. A Mod-FusedMBConv block is introduced into the early stage of EfficientNetV2-S to provide input-dependent local contextual modulation through a convolutional two-branch design.
2. The SE modules in the later MBConv stages are replaced with ECA, reducing attention-related parameters while retaining local cross-channel interaction.
3. The resulting architecture is evaluated through repeated runs, factorial component ablations, comparisons with general-purpose and mobile-oriented networks, paired statistical tests, and multidimensional efficiency measurements.

## 2 Related work

Deep-learning methods for visual recognition broadly include convolutional architectures, attention-based architectures, and hybrid designs. Standard self-attention explicitly models content-dependent pairwise interactions among tokens, but global attention may introduce substantial computational and memory costs as the number of tokens increases. Convolution-based contextual modulation offers an alternative design route by combining convolutional context aggregation with input-dependent feature transformation without explicitly computing pairwise token interactions.

### 2.1 Traditional feature engineering for unsound wheat grain classification

To identify unsound grains in wheat, traditional feature-engineering-based classification methods require researchers to manually extract key features, such as color and texture. For example, some researchers employed THz time-domain spectroscopy and the SMOTE algorithm for feature extraction and processing, respectively, before using LS-SVM for classification (Yuan et al., 2023). Similarly, in the study, researchers captured wheat seed images using linear charge-coupled devices (CCDs), extracted features using specialized software, and built models using traditional machine learning methods such as PLS-DA and SVMDA (Chen et al., 2012).Some utilized handcrafted shape and color features combined with a two-layer backpropagation neural network to classify unsound wheat, achieving a 2.5% error rate, reflecting the effectiveness of traditional feature engineering methods (Cheng et al., 2010).

Although classic feature-engineering-based techniques have shown positive results in identifying unsound wheat grains, the extracted features heavily rely on manually defined attributes and are often too specific. This limits the generalization ability of these methods, and their lack of automatic learning capability affects the model’s stability and consistency.

### 2.2 Wheat grain classification based on deep learning

Deep CNNs reduce dependence on manually specified descriptors by learning task-specific representations directly from images. CNNs jointly optimize feature extraction and classification, allowing discriminative representations to be learned from labeled data. For example, some proposed a traditional CNN specifically for wheat classification based on ResNet (Gao et al., 2022). Some proposed a deep learning-based TLFF model that leverages transfer learning and multi-model feature fusion to address limited data challenges in unsound wheat classification, significantly outperforming traditional methods (Zhang et al., 2022). Similarly, some improved YOLOv5 and applied the modified model to unsound grain classification (Zhang et al., 2023). However, these CNN-based methods primarily rely on fixed convolutional operators and do not explicitly incorporate input-dependent feature modulation within individual blocks. This consideration motivates the investigation of lightweight contextual modulation in convolutional architectures.

With the emergence of Vision Transformer (ViT) in the field of computer vision, many researchers have applied it to downstream tasks such as unsound grain classification. For example, some used ViT for wheat unsound grain classification in their study, achieving an excellent accuracy of 98.61% (Çolak et al., 2024). However, as mentioned earlier, ViT inherently requires high computational resources, which makes it less friendly for small edge devices.

These studies motivate the development of compact architectures that balance classification accuracy, parameter count, and computational cost.

### 2.3 EfficientNetV2 as the baseline architecture

**Figure 1:**
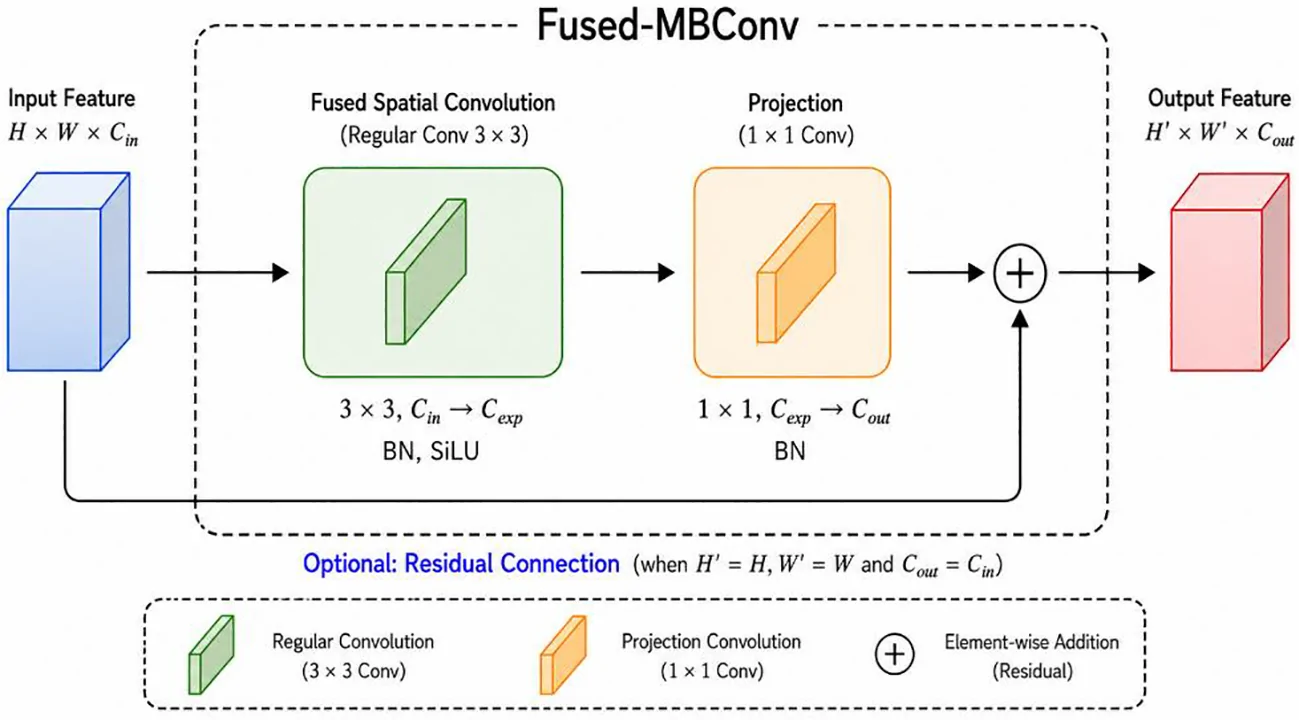
Fused-MBConv block.

Unlike earlier networks such as ResNet (He et al., 2016), which relied on deep stacking to improve classification accuracy, recent architecture design has increasingly emphasized the joint optimization of predictive performance, training speed, and computational cost. EfficientNetV2 (Tan and Le, 2021) was proposed in this context. Compared with EfficientNet (Tan and Le, 2019), EfficientNetV2 introduces a revised stage design and progressive training strategy to improve parameter efficiency and training speed. Specifically, the network’s early stage adopts the Fused-MBConv block to reduce computational overhead and accelerate inference; in the later stage, it continues using the MBConv block. This design helps keep the architecture compact while enhancing feature expression and generalization performance. The Fused-MBConv block is shown in **Figure 1**.

EfficientNetV2-S was selected as the baseline because it provides a structured combination of Fused-MBConv and MBConv stages within a moderate parameter budget. The early Fused-MBConv stage, however, does not contain an explicit input-dependent modulation branch. This study therefore investigates whether convolutional modulation can enrich early-stage representation without substantially increasing model complexity. We therefore redesign the early-stage block to incorporate input-dependent local contextual modulation.

### 2.4 Efficient convolutional modulation

Recent studies have explored convolutional modulation as a computationally efficient means of introducing input-dependent feature transformation into convolutional networks. Representative architectures such as FocalNet (Yang et al., 2022) and VAN (Guo et al., 2023) combine convolutional context aggregation with multiplicative feature modulation without explicitly computing pairwise token interactions.

**Figure 2:**
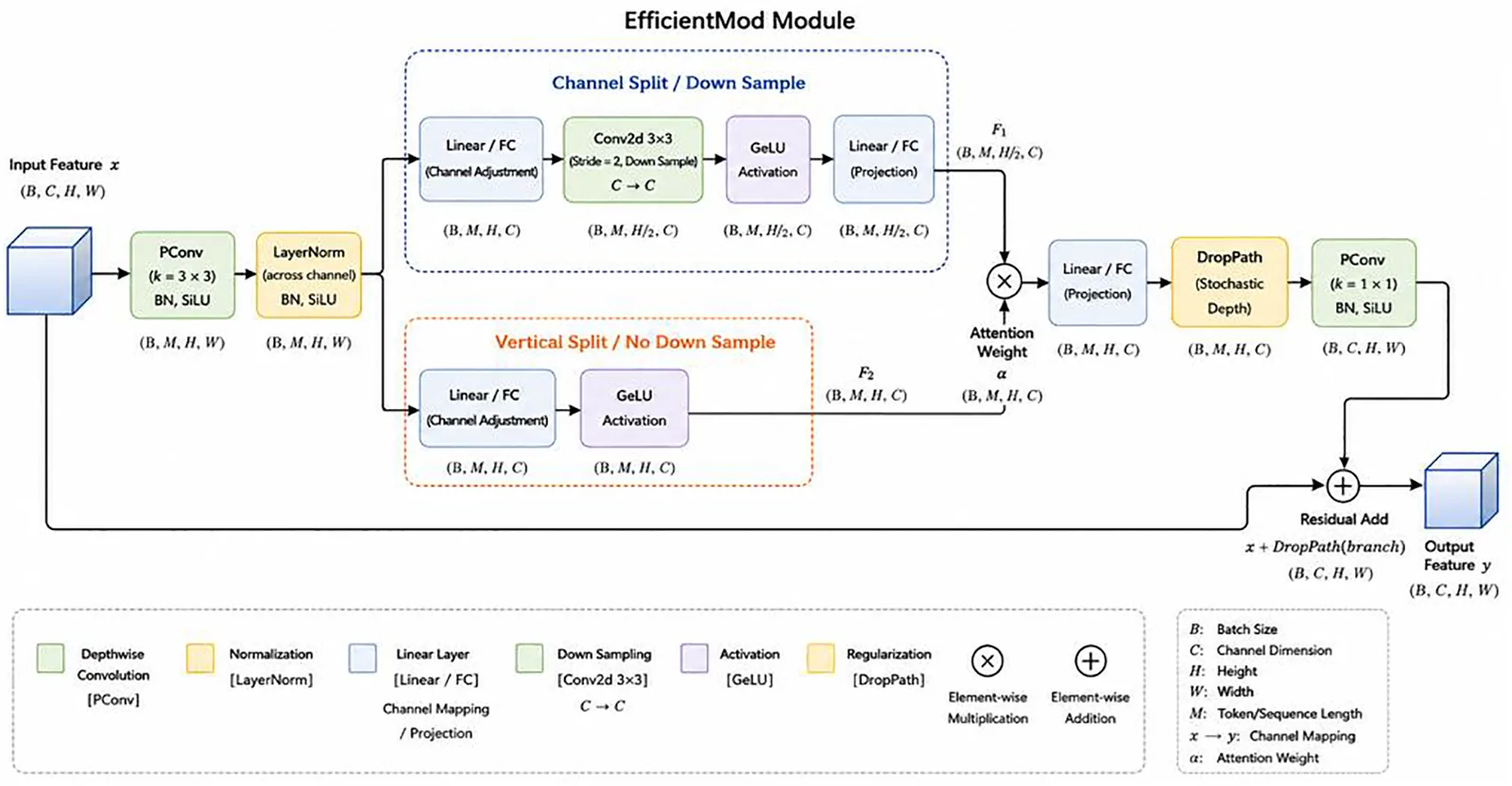
EfficientMod block.

Researchers reexamined the modulation mechanisms in FocalNet and VAN, finding that although these methods theoretically have lower parameter counts and fewer FLOPs, providing satisfactory performance, their inference speed still faces bottlenecks in resource-constrained environments (Ma et al., 2024). To address these issues, they proposed an efficient visual network design—Efficient Modulation. The structure is shown in **Figure 2**.

The above studies demonstrate that convolution-based modulation can introduce input-dependent feature transformation while retaining a convolutional computation graph. Such mechanisms do not explicitly reproduce the pairwise token-interaction mechanism of self-attention. Their convolution-based and relatively non-fragmented formulations provide useful references for introducing feature modulation into CNN architectures.

### 2.5 Incorporating channel attention mechanism into convolutional blocks

In recent years, incorporating channel attention mechanisms into convolutional blocks has gained widespread attention, mainly due to their significant role in improving parameter efficiency and feature representation.

One of the most representative blocks is the Squeeze-and-Excitation (SE) block (Hu et al., 2018), which learns channel attention for each convolution block through squeezing and excitation mechanisms, significantly improving the performance of deep CNN architectures.

**Figure 3:**
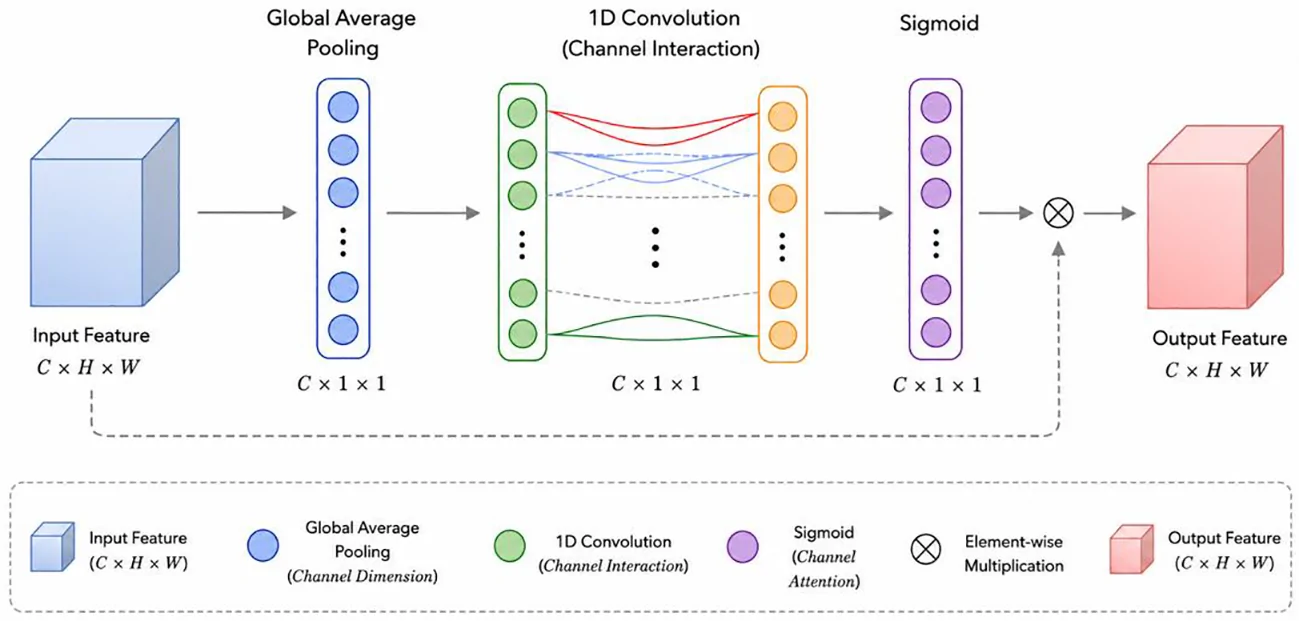
ECA block.

However, research has shown that the dimensionality reduction operation in the SE block may affect the accuracy of channel attention, and capturing the dependencies across all channels is both inefficient and redundant. To address this issue, the Efficient Channel Attention (ECA) block (Wang et al., 2020) was introduced. ECA models local cross-channel dependencies using a one-dimensional convolution without dimensionality reduction and introduces negligible parameter overhead. The structure of the ECA block is shown in **Figure 3**.

ECA therefore provides a parameter-efficient alternative to SE for modeling local cross-channel interactions without dimensionality reduction.

## 3 Proposed method

To improve the accuracy–parameter trade-off of EfficientNetV2-S, we propose ECA-ModNet. The network modifies the early Fused-MBConv stage using input-dependent local contextual modulation and replaces the later SE modules with ECA to reduce attention-related parameters.

### 3.1 Proposed Mod-FusedMBConv block

To introduce input-dependent local contextual modulation into the early stage of EfficientNetV2-S, the original Fused-MBConv block is replaced with the proposed Mod-FusedMBConv block.

The proposed Mod-FusedMBConv block consists of a context branch and a modulation branch. The context branch aggregates local spatial information, whereas the modulation branch generates input-dependent modulation features. Their outputs are combined by element-wise multiplication and projected back to the original channel dimension. The core operation is formulated in Equation 1:

(1) f=ctx(x)·v(x)

**Figure 4:**
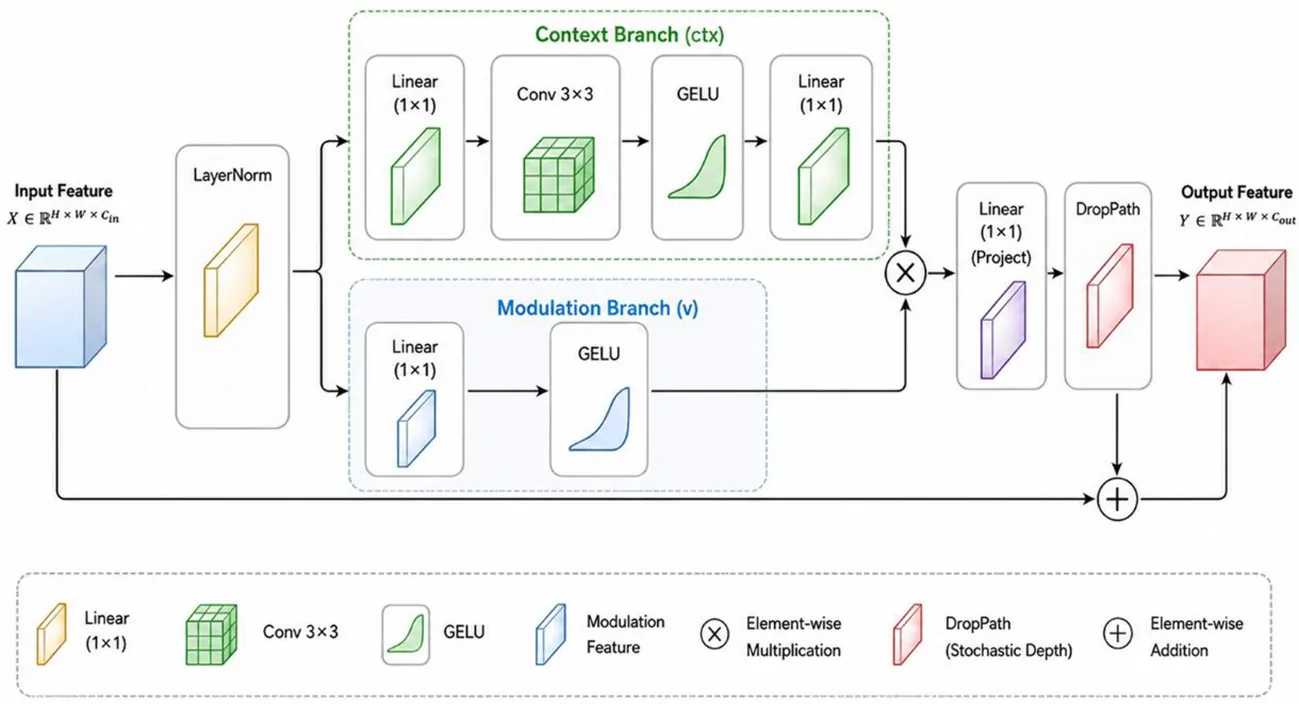
Mod-FusedMBConv block.

The two parallel branches transform the normalized input from complementary perspectives. The context branch, *ctx*(*x*), captures local spatial patterns through channel projection and a standard 3×3 convolution. The modulation branch, *v*(*x*), generates input-dependent features that adaptively recalibrate the context representation. The two outputs are combined through element-wise multiplication, followed by output projection and residual aggregation. Because the context branch uses a standard 3 × 3 convolution, the proposed block performs local context aggregation and does not explicitly model pairwise global dependencies as self-attention does. This design introduces input-dependent feature modulation while retaining a convolution-based computation graph. **Figure 4** illustrates the architecture of Mod-FusedMBConv.

The input tensor is first converted from NCHW to NHWC because LayerNorm and the linear projections operate along the last dimension. In the context branch, the tensor is converted back to NCHW before the standard 3×3 convolution and then returned to NHWC for activation and linear projection. The modulated feature is projected, regularized using DropPath, converted back to NCHW format, and added to the residual input. The definitions of the operators used in **Algorithm 1** are summarized in **Table 1**.

Algorithm 1 **Pseudocode of the Mod-FusedMBConv block.**

# Input/output shape: (B, C, H, W); batch size B; Feature map height H, width W, dim Cdef init():   ctx_fc1 = Linear(C, C)   ctx_conv = Conv2d(C, C, kernel_size=3, stride=1, padding=1)   ctx_fc2 = Linear(C, C)   v = Sequential(Linear(C, C), GELU())   proj = Linear(C, C)def forward(x):   x0 = LayerNorm(x.permute(0, 2, 3, 1))   x1 = ctx_fc1(x0)   x1 = x1.permute(0, 3, 1, 2)   x1 = ctx_conv(x1)   x1 = x1.permute(0, 2, 3, 1)   x1 = ctx_fc2(GELU(x1))   x2 = v(x0)   x1 = proj(x1 * x2)   x1 = DropPath(x1)return x + x1.permute(0, 3, 1, 2)

**Table 1:** Definitions of operators used in **Algorithm 1**.

Variables	Description
Linear	Linear transformation
Conv2d (C, C, kernel_size=3, stride=1, padding=1)	Standard 3×3 convolution performed in NCHW format
Sequential	A container that processes input sequentially
GELU	Activation function
LayerNorm	Layer normalization
Permute(0, 2, 3, 1)	Converts NCHW to NHWC
Permute(0, 3, 1, 2)	Converts NHWC to NCHW
DropPath	A regularization method

In the context branch, a standard 3×3 convolution is used to aggregate local spatial information. Linear projections are applied before and after the convolution to transform the channel representation and align it with the modulation branch. The context transformation is defined in Equation 2:

(2) $$ctx\left(\text{x}\right)=Linear\left(GeLU\left({\text{Conv}}_{3\times 3}\left(\text{Linear}\left(\text{x}\right)\right)\right)\right)$$

In the *v*(*x*) branch, a linear projection followed by GELU activation maps the normalized input into a modulation feature space, as expressed in Equation 3:

(3) v(x)=GELU(Linear(x))

The outputs of the context and modulation branches are first combined through element-wise multiplication, followed by a linear projection, as shown in Equation 4:

(4) m=Linear(ctx(x)·v(x))

The projected feature is then regularized by DropPath and combined with the input through a residual connection.

Compared with the original Fused-MBConv block, Mod-FusedMBConv introduces input-dependent feature modulation through the interaction between the context and modulation branches. The context branch captures local spatial patterns, whereas the modulation branch provides adaptive feature responses derived from the normalized input. The multiplicative interaction produces an input-dependent feature response before output projection and residual aggregation.

### 3.2 Improved MBConv with ECA

In the later MBConv stages, SE is replaced with ECA to reduce attention-related parameters and retain local cross-channel interaction without dimensionality reduction.

ECA avoids channel dimensionality reduction and models local cross-channel interactions using a one-dimensional convolution. Compared with the fully connected bottleneck in SE, this formulation requires substantially fewer attention-related parameters.

**Figure 5:**
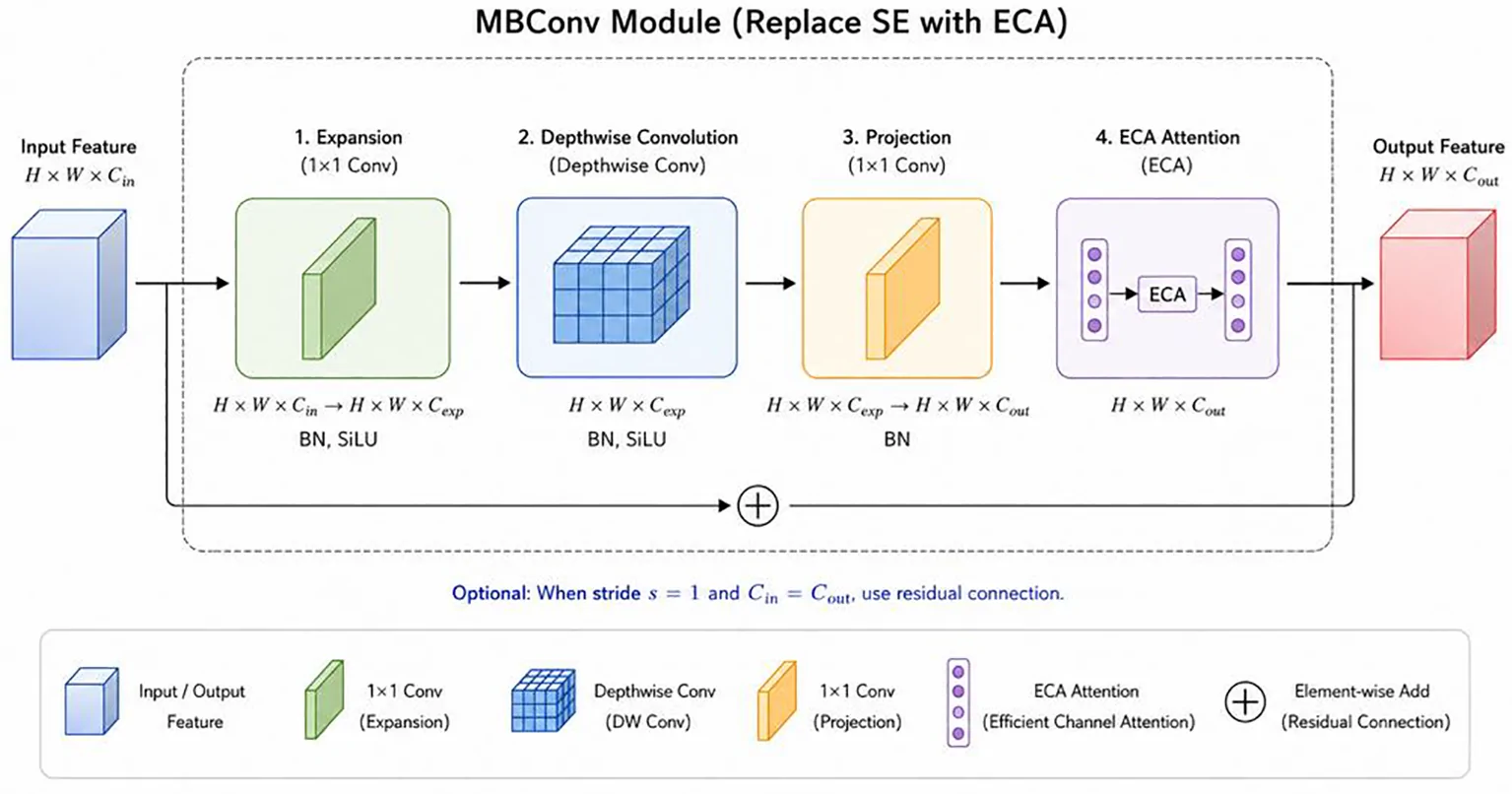
Improved MBConv block.

In the proposed ECA-MBConv block, the original SE module is removed, and the ECA module is applied after the projection convolution, as illustrated in **Figure 5**. Let $X\in R^{B\times C\times H\times W}$ denote the feature tensor produced by the projection convolution, where *B*, *C*, *H*, and *W* represent the batch size, number of channels, height, and width, respectively. ECA first applies global average pooling to aggregate the spatial information of each channel, as defined in Equation 5:

(5) $$z_{b,c}=\frac{1}{HW}\sum_{i=1}^{H}\sum_{j=1}^{W}X_{b,c,i,j},$$

where $z=GAP\left(X\right)\in {\mathbb{R}}^{B\times C}$ denotes the channel descriptor. A one-dimensional convolution with kernel size *k* is subsequently applied along the channel dimension, followed by a sigmoid activation, as shown in Equation 6:

(6) $$\omega =\sigma \left(Conv1D_{k}\left(z\right)\right),$$

where $\omega \in R^{B\times C}$ denotes the learned channel attention weights. Finally, the weights are broadcast along the spatial dimensions and multiplied with the input feature tensor, as expressed in Equation 7:

(7) $$Y_{b,c,i,j}=X_{b,c,i,j}\omega_{b,c},$$

which can be compactly expressed as Equation 8:

(8) Y=X⊙ω.

Here, ⊙ denotes channel-wise multiplication with spatial broadcasting.

Replacing SE with ECA reduces the number of attention-related parameters while preserving channel-wise recalibration. The structure of the improved MBConv Block is shown in **Figure 5**.

### 3.3 Proposed ECA-ModNet

ECA-ModNet is constructed by integrating Mod-FusedMBConv and ECA-MBConv into selected stages of EfficientNetV2-S. The two compatible Fused-MBConv blocks in Stage 1 are replaced with Mod-FusedMBConv blocks to introduce input-dependent local contextual modulation. In Stages 4–6, the original SE modules are replaced with ECA to reduce attention-related parameters while preserving cross-channel interaction. The remaining stages retain the original EfficientNetV2-S configuration. **Table 2** summarizes the ECA-ModNet architecture.

**Table 2:** ECA-ModNet architecture.

Stage	Operator	Stride	Channels	Layers
0	Conv3×3	2	24	1
1	Mod-FusedMBConv	1	24	2
2	Fused-MBConv	2	48	4
3	Fused-MBConv	2	64	4
4	ECA-MBConv	2	128	6
5	ECA-MBConv	1	160	9
6	ECA-MBConv	2	256	15
7	Conv1×1& Pooling&FC	–	1280	1

ECA-ModNet retains the overall stage organization of EfficientNetV2-S while modifying selected blocks in the early and later stages. Under an input resolution of 224×224, the resulting network contains 16.49M parameters and requires 2.95G FLOPs. This configuration retains the stage structure of EfficientNetV2-S while modifying the early contextual interaction and later channel-attention mechanisms.

## 4 Experiments

### 4.1 Dataset selection and sample construction

Experiments were conducted using the publicly available GrainSpace dataset (Fan et al., 2022). GrainSpace contains approximately 5.25 million images of wheat, maize, and rice acquired using three imaging devices across eight regions in five countries. The images were annotated by trained grain-quality inspectors. The present study used the G600 wheat subset because it provides single-kernel images with clearly visible color, texture, and morphological characteristics.

This study selected wheat kernel images captured using the G600 device. The G600 subset contains single-kernel images with relatively clear texture, color, and morphological information. Seven wheat kernel quality categories were included: normal kernels (NOR), kernels attacked by pests (AP), broken kernels (BN), black-point kernels (BP), Fusarium-infected and shriveled kernels (FS), moldy kernels (MY), and sprouted kernels (SD). The classification task comprised one normal class and six unsound-kernel classes.

**Figure 6:**
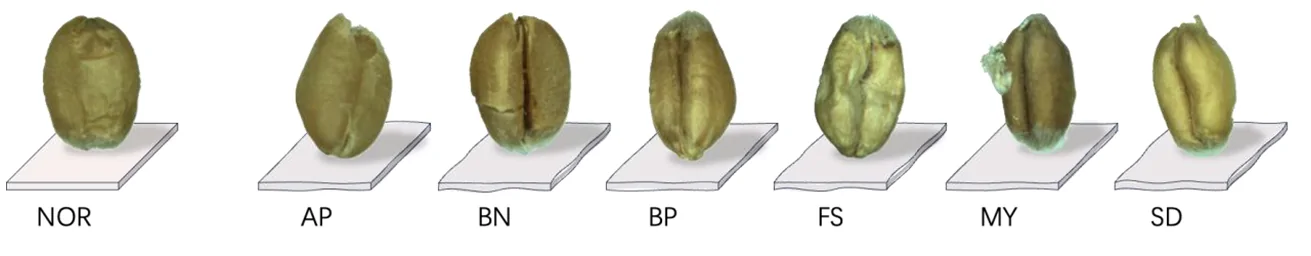
Representative samples of the seven wheat kernel quality categories selected from the G600 subset of GrainSpace.

Each selected image depicts a single physical wheat kernel. To construct a class-balanced dataset, 3,600 images were randomly sampled from all available images in each category using a fixed random seed of 42. The resulting dataset contained 25,200 images. Representative samples from the seven categories are shown in **Figure 6**.

### 4.2 Dataset splitting and image processing

**Figure 7:**
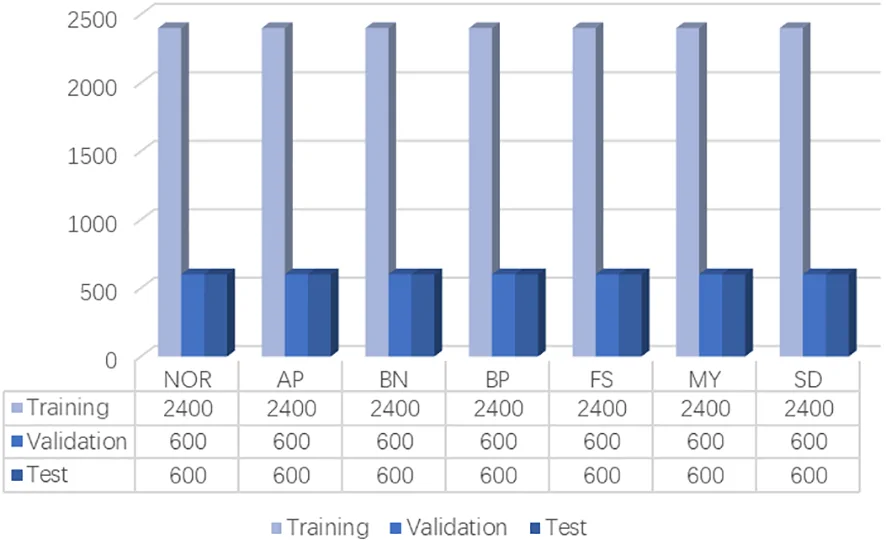
Distribution of training set, validation set, and test set.

A class-stratified random splitting strategy was adopted to construct the training, validation, and test sets. For each of the seven categories, the 3,600 selected images were randomly divided at a ratio of 4:1:1 using a fixed random seed of 42. Specifically, each category contained 2,400 training images, 600 validation images, and 600 test images. The resulting training, validation, and test sets contained 16,800, 4,200, and 4,200 images, respectively. The class-wise distribution is shown in **Figure 7**.

Because each G600 image corresponds to a distinct physical wheat kernel, images of the same physical kernel were not shared across the three subsets. Dataset partitioning was completed before any image augmentation was performed. Because partitioning preceded augmentation, no augmented derivative of a training image was included in the validation or test set.

The same fixed split was used for ECA-ModNet, all ablation variants, and all comparison models. The training set was used for model parameter optimization, whereas the validation set was used for checkpoint selection and hyperparameter tuning. The test set was reserved exclusively for final evaluation.

**Figure 8:**
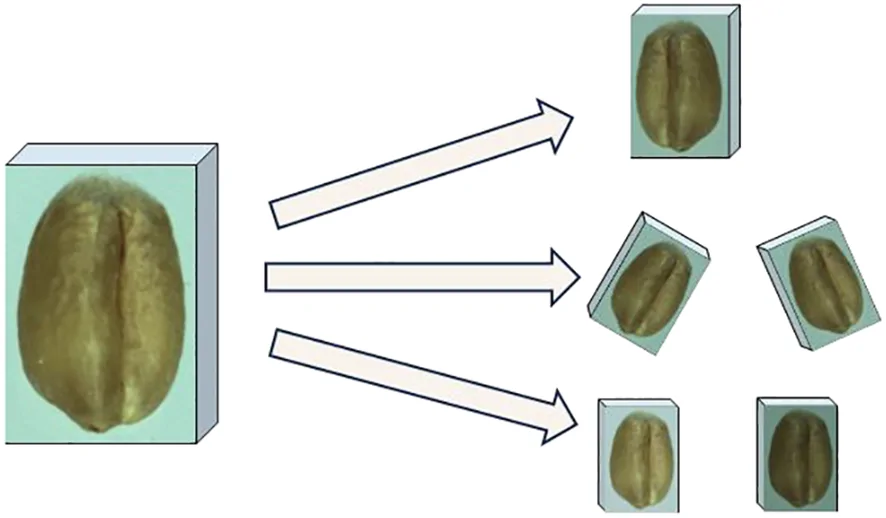
Data augmentation examples.

Online augmentation was applied only to the training set. Each image was randomly resized and cropped to 224×224, horizontally flipped with a probability of 0.5, rotated within ±30° with a probability of 0.5, and subjected to random brightness scaling within [0.8,1.2]. Validation and test images were processed using deterministic resizing, center cropping, tensor conversion, and normalization. Representative augmented images are shown in **Figure 8**.

### 4.3 Experimental protocol and evaluation methods

Model training, complexity profiling, and desktop runtime benchmarking were conducted on a workstation equipped with an Intel Core i7-12700H processor and an NVIDIA GeForce RTX 3060 GPU running Windows 11. The models were implemented in PyTorch. Embedded deployment benchmarking was conducted separately on an NVIDIA Jetson Orin Nano Super using FP16 TensorRT inference, as described in Section 4.4.4. Models evaluated on the same platform used identical software and inference settings.

All models used the fixed training, validation, and test split described in Section 4.2. Official ImageNet-1K pretrained weights were used to initialize the feature-extraction backbones. The original classification heads were replaced with seven-class classifiers. Newly introduced layers, including the ECA and Mod-FusedMBConv components, were initialized separately. Input images were processed at a resolution of 224×224 pixels. Stochastic augmentation was applied only to the training set, whereas the validation and test sets underwent identical deterministic preprocessing.

Each model was trained for 150 epochs using stochastic gradient descent. The initial learning rate was set to 0.01, and the weight decay was 1×10^−4^. A cosine annealing schedule was used to adjust the learning rate during training. Cross-entropy loss served as the optimization objective, and the batch size was set to 128. Training used automatic mixed precision. The main training settings are summarized in **Table 3**.

**Table 3:** Experimental parameters setting.

Parameter	Value or name
Input image size	224×224
Batch size	128
Training epochs	150
Learning rate	0.01
Learning Rate Schedule	Cosine Annealing Learning Rate
Weight Decay	1e-4
Loss function	CrossEntropyLoss
Optimizer	Stochastic Gradient Descent

For each independent run, the checkpoint with the highest validation accuracy was retained. Model configuration, checkpoint selection, and hyperparameter determination were based exclusively on validation-set performance. The test set was not used for architecture selection or model tuning and was evaluated only after the final configuration had been fixed. Each experiment was independently repeated three times using random seeds 2024, 2025, and 2026. Unless otherwise stated, classification results are reported as the mean and sample standard deviation across independent runs.

Classification performance was evaluated using accuracy, macro-precision, macro-recall, and macro-F1. For class c, precision, recall, and F1-score were defined in Equations 9–11, respectively.

(9) $$P_{c}=\frac{TP_{c}}{TP_{c}+FP_{c}},$$

(10) $$R_{c}=\frac{TP_{c}}{TP_{c}+FN_{c}},$$

(11) $$F1_{c}=\frac{2P_{c}R_{c}}{P_{c}+R_{c}},$$

where, *TP_c_*, *FP_c_* and *FN_c_* denote the true-positive, false-positive, and false-negative counts for class c, respectively. The corresponding macro-averaged metrics were calculated by assigning equal weight to all C classes, as shown in Equations 12–14:

(12) $$\text{Macro-Precision}=\frac{1}{C}\sum_{c=1}^{C}P_{c},$$

(13) $$\text{Macro-Recall}=\frac{1}{C}\sum_{c=1}^{C}R_{c},$$

(14) $$\text{Macro-F}1=\frac{1}{C}\sum_{c=1}^{C}F1_{c}.$$

The balanced test set yielded numerically similar values for accuracy and macro-recall. Macro-precision and macro-F1 were additionally reported to characterize class-wise performance.

Model efficiency was assessed using parameter count, FLOPs, GPU latency, CPU latency, throughput, peak GPU memory, and GPU energy consumption per image. Efficiency was characterized by parameter count, FLOPs, GPU and CPU latency, throughput, peak GPU memory, and GPU energy consumption per image. FLOPs were computed for an input tensor of 1×3×224×224 using a consistent profiling convention across models.

Runtime measurements were obtained on the same workstation under identical inference settings. Each model was placed in evaluation mode, and gradient computation was disabled. GPU latency was measured with a batch size of one. Fifty warm-up iterations were performed before 300 timed forward passes. CUDA synchronization was applied during timing to account for asynchronous GPU execution. CPU latency was measured under the same input setting. Throughput was reported in images per second, and peak GPU memory was recorded after resetting the memory statistics. The same numerical precision was used for all models included in the efficiency comparison.

A positive value indicates higher accuracy for ECA-ModNet. The mean paired difference and its 95% confidence interval were calculated. Paired t-tests were used to evaluate whether the mean difference was zero. Because ECA-ModNet was compared with multiple models, the resulting p-values were adjusted using the Holm procedure to control the family-wise error rate. A Holm-adjusted *p* < 0.05 was considered statistically significant. Statistical conclusions were drawn jointly from the adjusted p-values, confidence intervals, and observed difference magnitudes.

### 4.4 Comparison experiments

ECA-ModNet was compared with representative general-purpose and lightweight architectures. The general-purpose models included ResNet-50 (He et al., 2016), DenseNet-169 (Huang et al., 2017), EfficientNet-B4 (Tan and Le, 2019), RepVGG-A2 (Ding et al., 2021), ConvNeXt-Tiny (Liu et al., 2022), InceptionNeXt-T (Yu et al., 2024), and EfficientNetV2-S (Tan and Le, 2021). The lightweight models included MobileNetV3-Large (Howard et al., 2019), ShuffleNetV2 1.0× (Ma et al., 2018), GhostNet 1.0× (Han et al., 2020), EfficientNet-Lite0 (Tan and Le, 2019), MobileViT-XS (Mehta and Rastegari, 2021), and RepViT-M1.0 (Wang et al., 2024). All models were trained and evaluated using the experimental protocol described in Section 4.3.

#### 4.4.1 Classification performance

The classification results are presented in **Table 4**. ECA-ModNet achieved an accuracy of 90.17 ± 0.21% and a macro-F1 score of 90.23 ± 0.21%, both of which were the highest mean values among the evaluated models. Relative to EfficientNetV2-S, ECA-ModNet improved accuracy from 86.37 ± 0.35% to 90.17 ± 0.21% and macro-F1 from 86.39 ± 0.35% to 90.23 ± 0.21%. These gains corresponded to 3.80 and 3.84 percentage points, respectively.

**Table 4:** Classification performance comparison with general-purpose and lightweight networks.

Model	Accuracy (%)	Macro-precision (%)	Macro-recall (%)	Macro-F1 (%)
ResNet-50	81.18 ± 0.06	81.35 ± 0.03	81.18 ± 0.06	81.23 ± 0.06
DenseNet-169	81.46 ± 0.41	81.65 ± 0.38	81.46 ± 0.41	81.52 ± 0.39
EfficientNet-B4	85.42 ± 0.31	85.40 ± 0.37	85.42 ± 0.31	85.57 ± 0.25
RepVGG-A2	83.43 ± 0.38	83.51 ± 0.38	83.43 ± 0.38	83.48 ± 0.36
ConvNeXt-Tiny	90.09 ± 0.14	89.95 ± 0.51	90.09 ± 0.14	89.81 ± 0.69
InceptionNeXt-T	90.10 ± 0.40	90.35 ± 0.44	90.10 ± 0.40	90.21 ± 0.45
MobileNetV3-Large	87.14 ± 0.19	87.29 ± 0.22	87.14 ± 0.19	87.19 ± 0.20
ShuffleNetV2 1.0x	86.48 ± 0.39	86.61 ± 0.47	86.48 ± 0.39	86.52 ± 0.42
GhostNet 1.0x	87.11 ± 0.44	87.39 ± 0.42	87.11 ± 0.44	87.19 ± 0.44
EfficientNet-Lite0	86.32 ± 0.54	86.46 ± 0.44	86.32 ± 0.54	86.37 ± 0.51
MobileViT-XS	85.62 ± 0.34	85.44 ± 0.29	85.62 ± 0.34	85.31 ± 0.30
RepViT-M1.0	85.75 ± 0.37	85.55 ± 0.48	85.75 ± 0.37	85.79 ± 0.40
EfficientNetV2-S	86.37 ± 0.35	86.53 ± 0.34	86.37 ± 0.35	86.39 ± 0.35
**ECA-ModNet**	**90.17 ± 0.21**	**90.20 ± 0.25**	**90.17 ± 0.21**	**90.23 ± 0.21**

Bold values indicate the results of the proposed ECA-ModNet model.

ConvNeXt-Tiny and InceptionNeXt-T achieved comparable classification performance, with accuracies of 90.09 ± 0.14% and 90.10 ± 0.40%, respectively. Their macro-F1 scores were 89.81 ± 0.69% and 90.21 ± 0.45%. The mean accuracy differences between these models and ECA-ModNet were 0.08 and 0.07 percentage points.

Among the lightweight models, MobileNetV3-Large and GhostNet 1.0× achieved the highest accuracies, reaching 87.14 ± 0.19% and 87.11 ± 0.44%, respectively. ShuffleNetV2 1.0× and EfficientNet-Lite0 obtained accuracies of 86.48 ± 0.39% and 86.32 ± 0.54%, while MobileViT-XS and RepViT-M1.0 achieved 85.62 ± 0.34% and 85.75 ± 0.37%. ECA-ModNet exceeded the six lightweight models by 3.03–4.55 percentage points in mean accuracy. The macro-F1 results followed the same overall pattern.

**Figure 9:**
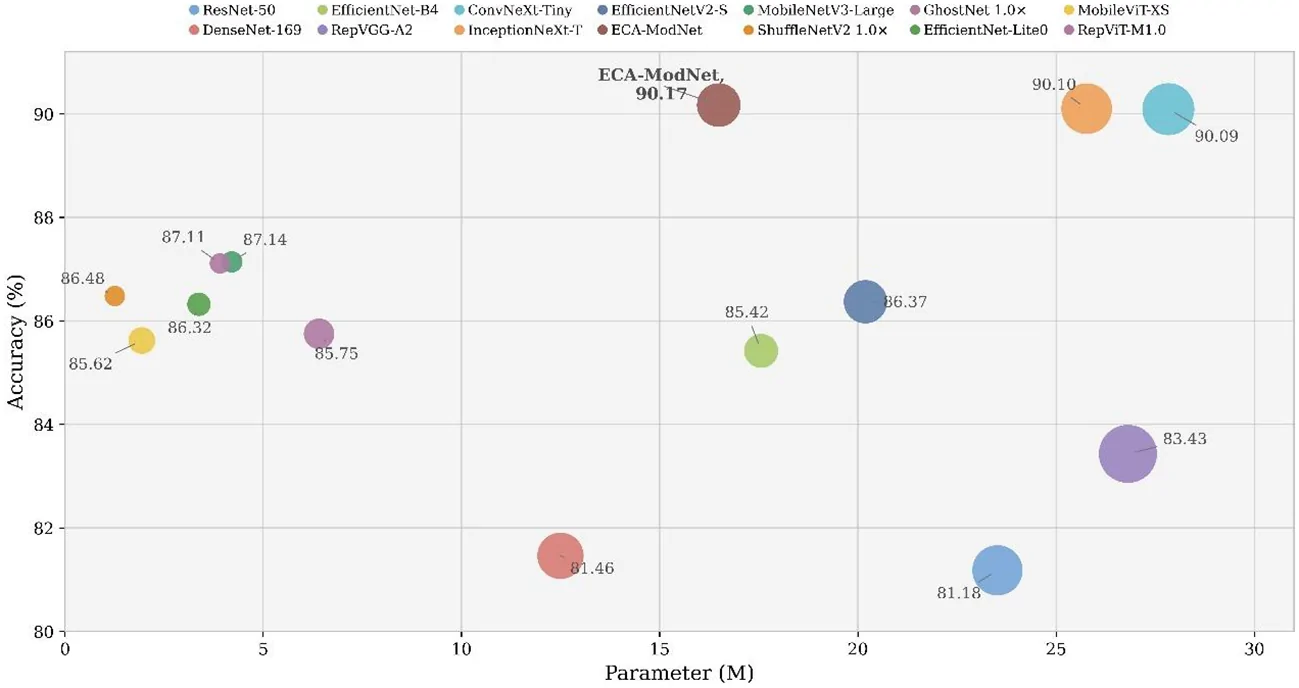
Accuracy–parameter comparison of all evaluated models. Bubble area is proportional to FLOPs.

**Figure 9** summarizes the relationship among classification accuracy, parameter count, and FLOPs. ECA-ModNet achieved 90.17% accuracy with 16.49 million parameters and 2.95 GFLOPs. ConvNeXt-Tiny and InceptionNeXt-T achieved similar accuracies but required 27.83M and 25.77M parameters, respectively. The lightweight models required substantially fewer parameters and FLOPs, although their classification accuracies remained below those of ECA-ModNet, ConvNeXt-Tiny, and InceptionNeXt-T.

**Figure 10:**
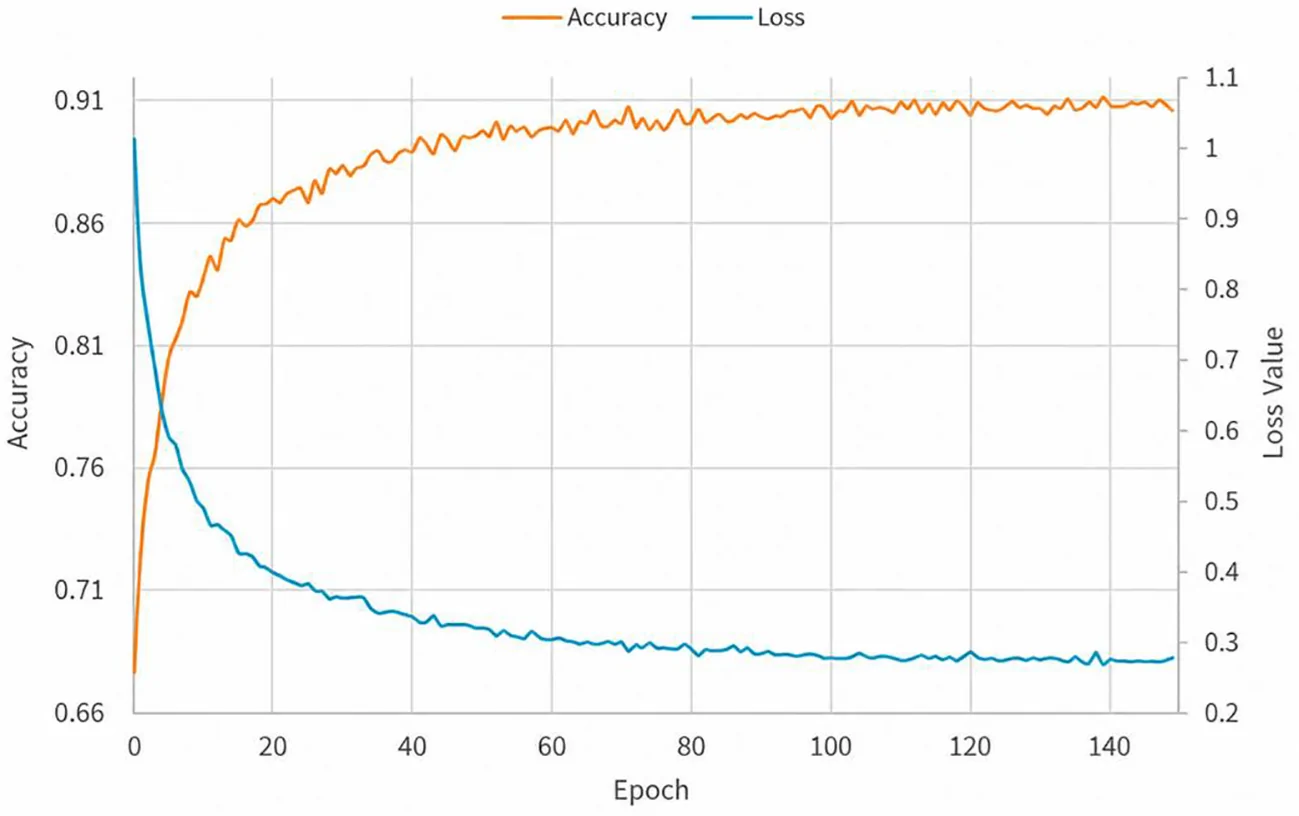
Accuracy and loss curve.

**Figure 10** shows the validation accuracy and loss curves of ECA-ModNet for a representative run. Validation accuracy increased rapidly during the early training stage and became stable after approximately 60 epochs. The validation loss showed a corresponding downward trend and remained stable during the later epochs. These curves indicate that the adopted optimization settings supported stable convergence.

#### 4.4.2 Computational efficiency comparison

**Table 5** summarizes the computational complexity and runtime performance of the evaluated models. ECA-ModNet contained 16.49M parameters, representing an 18.33% reduction relative to EfficientNetV2-S. Its FLOPs increased slightly from 2.90G to 2.95G, corresponding to an increase of 1.72%. The modification reduced the parameter count but slightly increased arithmetic complexity.

**Table 5:** Computational efficiency of different models.

Model	Params↓ (M)	FLOPs↓ (G)	GPU latency↓ (ms/image)	CPU latency↓ (ms/image)	Throughput↑ (images/s)	Peak GPU memory↓ (MB)	GPU energy↓ (J/image)
ResNet-50	23.52	4.11	10.56	52.26	94.71	343.76	0.615
DenseNet-169	12.50	3.40	36.44	120.32	27.44	185.96	1.552
EfficientNet-B4	17.56	1.54	27.74	81.88	36.05	259.21	0.993
RepVGG-A2	26.81	5.70	11.22	50.46	89.12	390.23	0.769
ConvNeXt-Tiny	27.83	4.47	13.08	45.87	76.44	505.16	0.802
InceptionNeXt-T	25.77	4.19	16.13	65.26	62.00	481.92	0.897
MobileNetV3-Large	4.21	0.22	20.27	30.47	49.33	74.41	0.586
ShuffleNetV2 1.0×	1.26	0.15	12.12	29.65	82.48	34.85	0.332
GhostNet 1.0×	3.91	0.15	19.03	45.33	52.54	70.95	0.554
EfficientNet-Lite0	3.38	0.40	10.18	30.59	98.22	64.59	0.334
MobileViT-XS	1.94	0.72	21.80	42.95	45.87	47.23	0.735
RepViT-M1.0	6.41	1.12	23.40	64.22	42.74	105.41	0.887
EfficientNetV2-S	20.19	2.90	11.92	49.48	83.90	295.98	0.677
**ECA-ModNet**	**16.49**	**2.95**	**13.43**	**63.75**	**74.46**	**379.87**	**0.667**

Bold values indicate the results of the proposed ECA-ModNet model.

ECA-ModNet required 13.43 ms per image on the GPU and 63.75 ms per image on the CPU, with a batch-1 throughput of 74.46 images/s. Compared with EfficientNetV2-S, its GPU and CPU latencies were 12.67% and 28.84% higher, respectively, while throughput was 11.25% lower. Peak GPU memory increased from 295.98 MB to 379.87 MB.

Relative to ConvNeXt-Tiny, ECA-ModNet reduced the parameter count by 40.75% and FLOPs by 34.00%. Its GPU latency was comparable, at 13.43 versus 13.08 ms per image, while peak GPU memory was 24.80% lower. Compared with InceptionNeXt-T, ECA-ModNet reduced the parameter count by 36.01% and FLOPs by 29.59%. It also achieved 16.74% lower GPU latency and 21.18% lower peak GPU memory.

The lightweight architectures remained more economical in model size and arithmetic complexity. ShuffleNetV2 1.0× contained only 1.26M parameters and required 0.15G FLOPs, while EfficientNet-Lite0 achieved the lowest GPU latency of 10.18 ms. Their lower computational cost was accompanied by lower classification accuracy than ECA-ModNet.

ECA-ModNet consumed 0.667 J per image, slightly lower than the 0.677 J per image of EfficientNetV2-S. Its energy consumption was also lower than those of ConvNeXt-Tiny and InceptionNeXt-T, which required 0.802 and 0.897 J per image, respectively. Several lightweight models remained more energy-efficient, particularly ShuffleNetV2 1.0× and EfficientNet-Lite0, with energy consumptions of 0.332 and 0.334 J per image.

ECA-ModNet provided a favorable accuracy–parameter trade-off relative to the high-accuracy general-purpose networks. Mobile-oriented architectures, however, retained clear advantages in parameter count, FLOPs, latency, memory usage, and energy consumption.

#### 4.4.3 Statistical significance analysis

**Figure 11:**
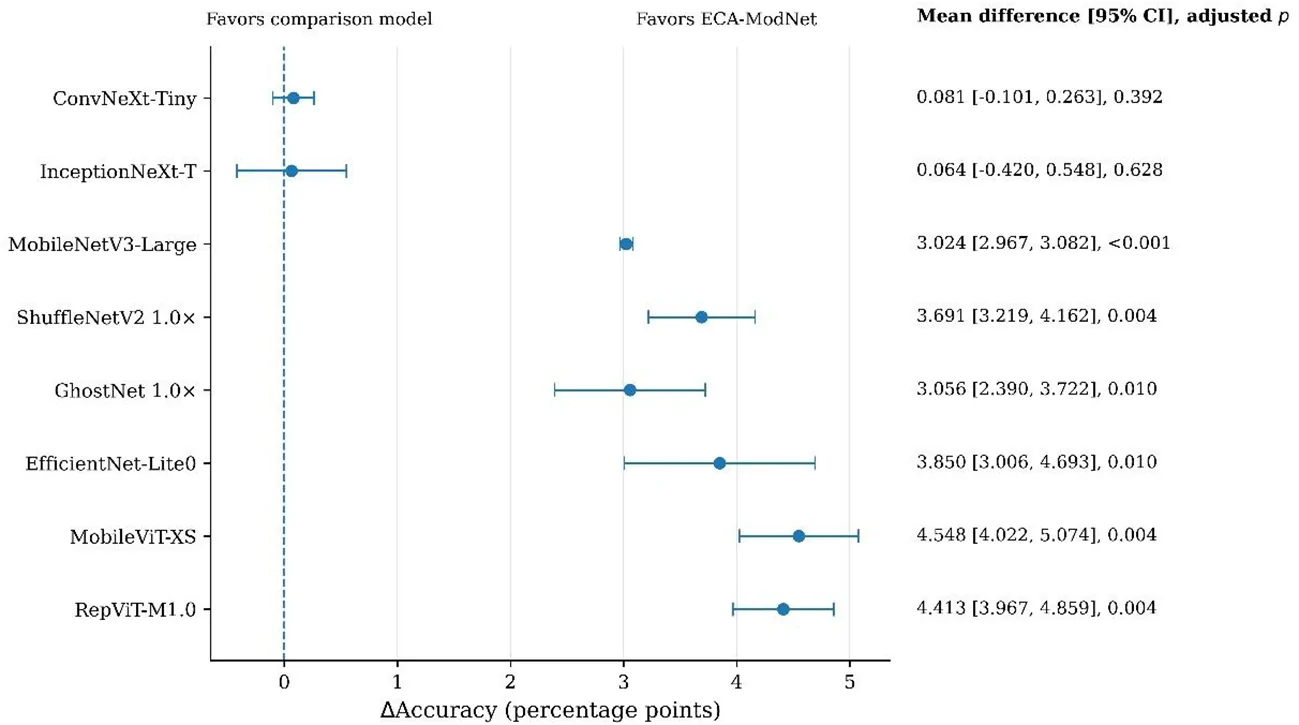
Forest plot of paired accuracy differences between ECA-ModNet and the representative comparison models.

**Table 6** presents the paired accuracy differences, 95% confidence intervals, and Holm-adjusted p-values, while **Figure 11** shows the corresponding forest plot. The mean accuracy difference between ECA-ModNet and ConvNeXt-Tiny was 0.081 percentage points, with a 95% confidence interval of [−0.101, 0.263] and an adjusted p-value of 0.392. The difference between ECA-ModNet and InceptionNeXt-T was 0.064 percentage points, with a 95% confidence interval of [−0.420, 0.548] and an adjusted p-value of 0.628. Neither comparison reached statistical significance.

**Table 6:** Statistical comparison between ECA-ModNet and representative models.

Model	Δ Accuracy (pp)	95% CI	Holm-adjusted *p*	Statistical interpretation
ConvNeXt-Tiny	0.081	[−0.101, 0.263]	0.392	No significant difference
InceptionNeXt-T	0.064	[−0.420, 0.548]	0.628	No significant difference
MobileNetV3-Large	3.024	[2.967, 3.082]	<0.001	ECA-ModNet significantly higher
ShuffleNetV2 ×1.0	3.691	[3.219, 4.162]	0.004	ECA-ModNet significantly higher
GhostNet	3.056	[2.390, 3.722]	0.010	ECA-ModNet significantly higher
EfficientNet-Lite0	3.850	[3.006, 4.693]	0.010	ECA-ModNet significantly higher
MobileViT-XS	4.548	[4.022, 5.074]	0.004	ECA-ModNet significantly higher
RepViT-M1.0	4.413	[3.967, 4.859]	0.004	ECA-ModNet significantly higher

ECA-ModNet achieved significantly higher accuracy than all six lightweight models. The mean differences ranged from 3.024 percentage points relative to MobileNetV3-Large to 4.548 percentage points relative to MobileViT-XS. All corresponding confidence intervals were entirely above zero, and the Holm-adjusted p-values were below 0.05.

ECA-ModNet did not differ significantly in accuracy from ConvNeXt-Tiny or InceptionNeXt-T, but achieved significantly higher accuracy than the six lightweight baselines.

#### 4.4.4 Embedded deployment evaluation

Embedded inference was evaluated for ECA-ModNet, EfficientNetV2-S, MobileNetV3-Large, ShuffleNetV2 1.0×, and EfficientNet-Lite0 on an NVIDIA Jetson Orin Nano Super. All models were exported to ONNX and converted into FP16 TensorRT engines. The input size was fixed at 1×3×224×224, with a batch size of one. Each engine underwent 50 warm-up iterations followed by 500 timed forward passes. Image loading, decoding, and preprocessing were excluded from latency measurement.

FP16 classification accuracy was evaluated on the complete test set and is reported as the mean and standard deviation across three independently trained checkpoints. Peak memory was defined as the maximum device memory occupied during inference. Energy consumption per image was calculated from the average board power and inference latency, as shown in Equation (15):

(15) $$E_{\text{image}}=P_{\text{avg}}\times \frac{t_{\text{latency}}}{1000},$$

where *P*_avg_ denotes the average board power during inference and *t*_latency_ denotes the mean inference latency in milliseconds.

As presented in **Table 7**, ECA-ModNet achieved the highest FP16 accuracy of 90.02 ± 0.30%. The corresponding accuracies of EfficientNetV2-S, MobileNetV3-Large, ShuffleNetV2 1.0×, and EfficientNet-Lite0 were 86.14 ± 0.26%, 86.98 ± 0.28%, 86.27 ± 0.41%, and 86.13 ± 0.34%, respectively. ECA-ModNet exceeded these models by 3.04–3.89 percentage points.

**Table 7:** Measured FP16 TensorRT deployment performance on the NVIDIA Jetson Orin Nano Super.

Model	FP16 accuracy (%)	Latency (ms/image)	Peak memory (MB)	Energy (J/image)
MobileNetV3-Large	86.98 ± 0.28	5.64	240.0	0.067
ShuffleNetV2 1.0×	86.27 ± 0.41	**4.36**	**185.0**	**0.049**
EfficientNet-Lite0	86.13 ± 0.34	5.02	225.0	0.059
EfficientNetV2-S	86.14 ± 0.26	11.46	500.0	0.166
**ECA-ModNet**	**90.02 ± 0.30**	12.91	628.0	0.185

Bold values indicate the best value for each metric, i.e., the highest accuracy and the lowest latency, peak memory, and energy consumption.

ECA-ModNet required 12.91 ms per image, 628.0 MB of peak memory, and 0.185 J per image. EfficientNetV2-S achieved a latency of 11.46 ms, with peak memory usage of 500.0 MB and energy consumption of 0.166 J per image. Among the lightweight architectures, ShuffleNetV2 1.0× recorded the lowest latency, peak memory usage, and energy consumption, at 4.36 ms, 185.0 MB, and 0.049 J per image, respectively. MobileNetV3-Large and EfficientNet-Lite0 achieved latencies of 5.64 and 5.02 ms, with corresponding energy consumption values of 0.067 and 0.059 J per image.

ECA-ModNet achieved the highest accuracy, whereas the lightweight models required less inference time, memory, and energy.

### 4.5 Ablation studies

Ablation experiments evaluated the contributions of ECA and Mod-FusedMBConv and the sensitivity of performance to block placement, block number, and context-kernel size.

#### 4.5.1 Component-level ablation

**Table 8** presents the component-level ablation results. EfficientNetV2-S was used as the baseline. The ECA-only variant replaced the original SE modules with ECA while retaining the original Fused-MBConv stages. The Mod-only variant introduced Mod-FusedMBConv without modifying the SE modules. ECA-ModNet combined both modifications.

**Table 8:** Component-level ablation study of Mod-FusedMBConv and ECA.

Model	Mod- FusedMBConv	ECA	Accuracy (%)	Macro- precision (%)	Macro-recall (%)	Macro-F1 (%)
EfficientNetV2-S	×	×	86.37 ± 0.34	86.53 ± 0.34	86.37 ± 0.34	86.39 ± 0.35
ECA-only	×	✓	88.37 ± 0.25	88.21 ± 0.27	88.37 ± 0.25	88.38 ± 0.38
Mod-only	✓	×	88.83 ± 0.27	89.08 ± 0.07	88.83 ± 0.27	88.84 ± 0.35
**ECA-ModNet**	**√**	**√**	**90.17 ± 0.21**	**90.20 ± 0.25**	**90.17 ± 0.21**	**90.23 ± 0.21**

Bold values indicate the best classification performance among the evaluated ablation configurations.

The baseline achieved an accuracy of 86.37 ± 0.34% and a macro-F1 score of 86.39 ± 0.35%. Replacing SE with ECA increased these metrics to 88.37 ± 0.25% and 88.38 ± 0.38%, respectively. The corresponding gains were 2.00 and 1.99 percentage points.

Introducing Mod-FusedMBConv alone produced an accuracy of 88.83 ± 0.27% and a macro-F1 score of 88.84 ± 0.35%, exceeding the baseline by 2.46 and 2.45 percentage points. The complete ECA-ModNet configuration achieved the highest accuracy of 90.17 ± 0.21% and the highest macro-F1 score of 90.23 ± 0.21%. Relative to ECA-only and Mod-only, its accuracy increased by 1.80 and 1.34 percentage points, respectively.

#### 4.5.2 Effect of insertion position and number

**Table 9** reports the effect of the insertion position and number of Mod-FusedMBConv blocks. ECA was retained in the later MBConv stages for all configurations, while only the placement and number of Mod-FusedMBConv blocks were varied.

**Table 9:** Effect of Mod-FusedMBConv insertion position and number.

Model	Accuracy (%)	Macro-precision (%)	Macro-recall (%)	Macro-F1 (%)
No insertion (0 blocks)	88.37 ± 0.25	88.21 ± 0.27	88.37 ± 0.25	88.38 ± 0.38
Stage 1, one block (1 block)	86.88 ± 0.31	86.11 ± 0.28	86.88 ± 0.31	86.71 ± 0.34
Stage 1 (2 blocks)	90.17 ± 0.21	90.20 ± 0.25	90.17 ± 0.21	90.23 ± 0.21
Stage 2 (3 blocks)	88.07 ± 0.27	88.47 ± 0.33	88.07 ± 0.27	88.10 ± 0.29
Stage 3 (3 blocks)	86.95 ± 0.42	86.52 ± 0.37	86.95 ± 0.42	86.86 ± 0.45
Stages 1 + 2 (5 blocks)	85.36 ± 0.36	85.30 ± 0.41	85.36 ± 0.36	85.10 ± 0.38
All fused stages (8 blocks)	83.57 ± 0.48	83.66 ± 0.44	83.57 ± 0.48	83.43 ± 0.46

The configuration without Mod-FusedMBConv achieved an accuracy of 88.37 ± 0.25%. Replacing one block in Stage 1 reduced the accuracy to 86.88 ± 0.31%. Replacing both compatible blocks in Stage 1 increased the accuracy to 90.17 ± 0.21%, which was the highest mean accuracy among the evaluated configurations.

Placing three Mod-FusedMBConv blocks in Stage 2 or Stage 3 yielded accuracies of 88.07 ± 0.27% and 86.95 ± 0.42%, respectively. Extending the replacement to five blocks across Stages 1 and 2 reduced the accuracy to 85.36 ± 0.36%. Replacing all eight compatible blocks further decreased the accuracy to 83.57 ± 0.48%.

The highest performance was obtained by replacing the two compatible blocks in Stage 1. This configuration was retained in the final architecture based on validation-set performance.

#### 4.5.3 Effect of context kernel size

**Table 10** compares three kernel sizes in the context branch of Mod-FusedMBConv. The 3×3 kernel achieved an accuracy of 90.17 ± 0.21% and a macro-F1 score of 90.23 ± 0.21%.

**Table 10:** Effect of the context-branch convolution kernel size.

Kernel size	Accuracy (%)	Macro-precision (%)	Macro-recall (%)	Macro-F1 (%)
3 × 3	90.17 ± 0.21	90.20 ± 0.25	90.17 ± 0.21	90.23 ± 0.21
5 × 5	88.64 ± 0.24	88.89 ± 0.46	88.64 ± 0.24	88.71 ± 0.29
7 × 7	87.21 ± 0.33	87.26 ± 0.41	87.21 ± 0.33	87.07 ± 0.47

Increasing the kernel size to 5×5 reduced the accuracy to 88.64 ± 0.24% and the macro-F1 score to 88.71 ± 0.29%. With a 7×7 kernel, the accuracy and macro-F1 score decreased further to 87.21 ± 0.33% and 87.07 ± 0.47%, respectively. Macro-precision and macro-recall followed the same trend.

The 3×3 kernel yielded the highest mean values across the reported metrics and was retained in the final configuration.

### 4.6 Class-wise performance analysis

**Figure 12:**
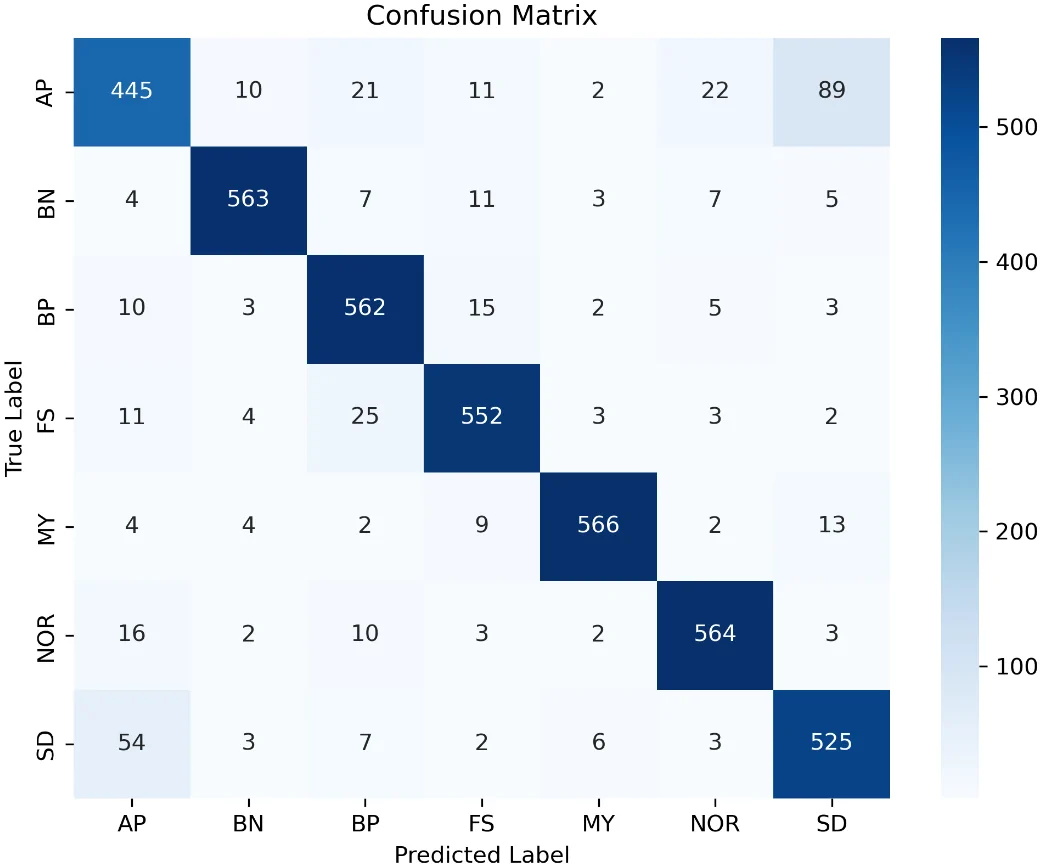
Confusion matrix.

**Figure 12** visualizes the confusion matrix for a representative run with a test accuracy of 89.93%, and **Table 11** reports the corresponding class-wise metrics. The model performed best on moldy, broken, and normal kernels, with F1-scores of 0.9561, 0.9470, and 0.9353, respectively. Performance was lower for pest-damaged and sprouted kernels, which yielded F1-scores of 0.7780 and 0.8468. The dominant confusion occurred between these two classes: 89 pest-damaged kernels were predicted as sprouted, and 54 sprouted kernels were predicted as pest-damaged. This error pattern suggests that their visible symptoms partially overlap in the current RGB images. More detailed local imaging, multi-view observations, or complementary spectral information may improve discrimination between these categories.

**Table 11:** Class-wise classification performance.

Class	Precision	Recall	F1-score
AP	0.8180	0.7417	0.7780
BN	0.9559	0.9383	0.9470
BP	0.8864	0.9367	0.9109
FS	0.9154	0.9200	0.9177
MY	0.9692	0.9433	0.9561
NOR	0.9307	0.9400	0.9353
SD	0.8203	0.8750	0.8468

### 4.7 Preliminary distillation analysis

To examine the effect of knowledge distillation (Hinton et al., 2015) on ECA-ModNet, EfficientNetV2-XL was employed as the teacher model and ECA-ModNet as the student model under a conventional logit-based distillation framework. The teacher model was trained on the same dataset and its parameters were fixed during student training. The student was optimized using cross-entropy supervision from the ground-truth labels together with the Kullback–Leibler divergence between the temperature-scaled output distributions of the teacher and student models. Temperatures of *τ* = 4, 6, and 8 were evaluated under the same data partitioning and evaluation protocol described in Section 4.3.

**Table 12** presents the test accuracies obtained under different distillation settings. The ECA-ModNet model trained without distillation achieved an accuracy of 89.93%. With knowledge distillation, the accuracies were 89.98%, 91.17%, and 88.02% at τ = 4, 6, and 8, respectively.

**Table 12:** Accuracy under different distillation temperatures.

ECA-ModNet	w/o Distill	w/Distill (τ = 4)	w/Distill (τ = 6)	w/Distill (τ = 8)
Test accuracy (%)	89.93	89.98	91.17	88.02

Among the evaluated settings, τ = 6 yielded the highest observed accuracy, exceeding the non-distilled result by 1.24 percentage points. The observed accuracies varied non-monotonically across the three temperature settings.

The results provide an initial assessment of the applicability of knowledge distillation to ECA-ModNet, while repeated experiments are required to further characterize the stability of the observed differences.

## 5 Discussion

ECA-ModNet improves the accuracy–parameter trade-off of EfficientNetV2-S through complementary modifications to its early and late stages. Mod-FusedMBConv introduces input-dependent local contextual modulation through the interaction between contextual and modulation features, whereas ECA refines channel responses without the dimensionality-reduction bottleneck of SE. In the component-level ablation, Mod-FusedMBConv and ECA increased accuracy by 2.46 and 2.00 percentage points, respectively; their combination yielded an accuracy of 90.17 ± 0.21% and a macro-F1 score of 90.23 ± 0.21%. The full model achieved the highest mean accuracy, although the combined gain was smaller than the sum of the individual gains, suggesting that the two modifications provide partially overlapping contributions.

The effectiveness of Mod-FusedMBConv depended strongly on its placement. Replacing the two compatible blocks in Stage 1 produced the highest accuracy, whereas extending the modification to deeper stages progressively reduced performance. Early feature maps are likely to retain fine-grained information related to kernel contours, surface texture, discoloration, embryo regions, and localized damage. Modulation at this stage strengthens interactions among these cues before spatial detail is compressed into higher-level representations. Under the present architecture and dataset, the 3×3 kernel provided a more effective local aggregation scale than the larger alternatives.

ECA-ModNet occupies a distinct operating point between high-capacity general-purpose networks and strongly compressed lightweight architectures. Its mean accuracy was close to those of ConvNeXt-Tiny and InceptionNeXt-T, while its parameter count was lower by 40.75% and 36.01%, respectively. The evaluated lightweight networks required fewer parameters and FLOPs, but their mean accuracies were 3.03–4.55 percentage points lower. ECA-ModNet therefore retains comparatively strong feature representation within a moderate parameter budget. This operating point may be useful when recognition accuracy and parameter storage are prioritized over minimum latency or energy consumption.

The runtime results reveal that parameter efficiency does not translate directly into lower execution cost. Relative to EfficientNetV2-S, ECA-ModNet reduced the parameter count by 18.33%, while FLOPs increased slightly from 2.90G to 2.95G. The additional modulation pathway also increased desktop inference latency and peak memory usage because of its intermediate activations and operator structure. In contrast, ECA-ModNet required fewer parameters, fewer FLOPs, and less peak GPU memory than ConvNeXt-Tiny and InceptionNeXt-T at a similar level of classification accuracy. These results highlight the multidimensional nature of computational efficiency: storage complexity, arithmetic cost, activation memory, and hardware latency reflect different architectural properties.

The FP16 TensorRT measurements on the NVIDIA Jetson Orin Nano Super followed the same accuracy–efficiency pattern. ECA-ModNet achieved an accuracy of 90.02 ± 0.30%, with a latency of 12.91 ms per image, peak memory usage of 628.0 MB, and energy consumption of 0.185 J per image. The lightweight architectures operated with lower latency, memory demand, and energy consumption, whereas ECA-ModNet retained higher recognition accuracy. The embedded results reproduced the same accuracy–efficiency trade-off observed on the desktop platform.

Class-wise analysis showed that errors were concentrated primarily between pest-damaged and sprouted kernels. The confusion may reflect partial overlap in embryo appearance, surface irregularity, and localized color variation between the two categories. By contrast, moldy, broken, normal, black-point, and Fusarium-infected or shriveled kernels displayed more distinctive combinations of texture, color, and morphology. Further improvement in the difficult categories may therefore depend on more informative observations, such as localized high-resolution imaging, multiple views, or complementary spectral features, rather than a uniform increase in model capacity.

The current study used a balanced subset acquired with the G600 imaging device. Further validation across imaging systems, wheat varieties, production regions, grain batches, and storage conditions is required to characterize cross-domain robustness. Runtime performance was evaluated on one desktop GPU and one embedded device. Future studies should evaluate heterogeneous edge hardware and examine quantization, structured pruning, operator fusion, and hardware-aware architecture refinement. End-to-end assessment in integrated grain-imaging and sorting systems should additionally include acquisition, preprocessing, inference, memory usage, energy consumption, sustained throughput, illumination variation, and kernel orientation.

## 6 Conclusion

This study presents ECA-ModNet, a parameter-efficient convolutional architecture for seven-class unsound wheat kernel classification. The network introduces input-dependent local contextual modulation into two early-stage Fused-MBConv blocks and replaces the later SE modules with ECA. Across three independent runs, ECA-ModNet achieved an accuracy of 90.17 ± 0.21% and a macro-F1 score of 90.23 ± 0.21%. Relative to EfficientNetV2-S, accuracy increased by 3.80 percentage points and the parameter count decreased from 20.19 million to 16.49 million, while computational cost increased marginally from 2.90 to 2.95 GFLOPs.

ECA-ModNet achieved accuracy statistically comparable to that of ConvNeXt-Tiny and InceptionNeXt-T with substantially fewer parameters, and significantly higher mean accuracy than the six lightweight baselines. Ablation experiments showed that two Stage 1 Mod-FusedMBConv blocks with a 3×3 context kernel yielded the highest mean performance among the evaluated configurations. These results indicate that ECA-ModNet provides a favorable trade-off between classification accuracy and parameter count. Further work should evaluate cross-device and cross-domain generalization, improve discrimination between visually similar kernel categories, and reduce runtime and memory costs on resource-constrained hardware.

## Data Availability

Publicly available datasets were analyzed in this study. This data can be found here: https://github.com/hellodfan/GrainSpace.
